# Long-living and highly efficient bio-hybrid light-emitting diodes with zero-thermal-quenching biophosphors

**DOI:** 10.1038/s41467-020-14559-8

**Published:** 2020-02-13

**Authors:** Anna Espasa, Martina Lang, Carmen F. Aguiño, Daniel Sanchez-deAlcazar, Juan P. Fernández-Blázquez, Uwe Sonnewald, Aitziber L. Cortajarena, Pedro B. Coto, Rubén D. Costa

**Affiliations:** 1 IMDEA Materials Institute, Calle Eric Kandel 2, 28906 Getafe, Spain; 2 Department of Biology, Friedrich-Alexander-University of Erlangen-Nuremberg, Staudtstraße 5, 91058 Erlangen, Germany; 3 Center for Cooperative Research in Biomaterials (CIC biomaGUNE), Basque Research and Technology Alliance (BRTA), Paseo de Miramon 182, 20014 Donostia-San Sebastián, Spain; 4 Ikerbasque, Basque Foundation for Science, María Diaz de Haro 3, 48013 Bilbao, Spain; 5 Department of Physical and Analytical Chemistry, University of Oviedo, Avenida Julián Clavería 8, 33006 Oviedo, Spain

**Keywords:** Biomaterials - proteins, Electronic devices, Organic LEDs

## Abstract

Bio-hybrid light-emitting diodes (Bio-HLEDs) based on color down-converting filters with fluorescent proteins (FPs) have achieved moderate efficiencies (50 lm/W) and stabilities (300 h) due to both thermal- and photo-degradation. Here, we present a significant enhancement in efficiency (*~*130 lm/W) and stability (>150 days) using a zero-thermal-quenching bio-phosphor design. This is achieved shielding the FP surface with a hydrophilic polymer allowing their homogenous integration into the network of a light-guiding and hydrophobic host polymer. We rationalize how the control of the mechanical and optical features of this bio-phosphor is paramount towards highly stable and efficient Bio-HLEDs, regardless of the operation conditions. This is validated by the relationships between the stiffness of the FP-polymer phosphor and the maximum temperature reached under device operation as well as the transmittance of the filters and device efficiency.

## Abstract (web summary)

Bio-hybrid LEDs (HLED) are an environmental friendly alternative to LEDs based on inorganic phosphors but achieving long term is challenging. Here, the authors present a long-living Bio-HLED based on a zero-thermal-quenching biophosphor design and investigate the photo-induced heat generation and dissipation processes.

## Introduction

In a few years, light-emitting diodes (LEDs) will rule artificial lighting for home, industry, display, and automotive sectors^1–5^. State-of-the-art devices consist of highly efficient blue LED chips covered by inorganic phosphors (IPs)—i.e., inorganic compounds doped with lanthanides (Ce-doped yttrium aluminum garnet (YAG:Ce) phosphors) and/or Cd-based quantum dots—that convert the blue light into a low-energy emission, rendering a white-emitting source. Although a myriad of IPs have been investigated, the lighting features demanded by real-life applications have only been achieved using toxic or rare-earth elements^6–8^. However, these IPs are now becoming a major concern because of (i) the visual and non-visual effects of the LED illumination on the human health, (ii) the environmental impact related to the IP production, and (iii) the lack of recycling protocols^9–12^.

These concerns have fueled the renaissance of hybrid LEDs (HLEDs)^13–15^. In these devices, the IPs are replaced by organic phosphors (OPs) ranging from polymer matrices doped with artificial emitters^16–18^, over luminescent metal organic frameworks (MOFs)^19–21^, to biophosphors based on either DNA/protein matrices to host artificial emitters^15,22–26^ or fluorescent proteins (FPs) embedded in polymer/MOF matrices or alone^15,27–34^. Like IPs, OPs must exhibit a narrow emission with high photoluminescence quantum yields (*ϕ*), high molar extinction coefficients (*ε*), outstanding photobleaching stabilities under operating conditions, and low heat generation and thermal quenching upon continuous excitation.

Irrespective of the nature of the phosphor (organic or inorganic), the most critical aspect for the development of long-lived devices is the photo-induced heat generation^13–15,35–38^. In this respect, the lack of a solid understanding of the heat generation and dissipation processes at device operating conditions has limited the number of IPs commercially available, though recent promising advances have been reported^35–37^. In OPs, the increase in temperature upon excitation at high fluencies promotes a fast photo-oxidation that drastically reduces the lifetime of the OPs^13–15^. As a consequence, only a handful of examples have shown stabilities over 1000 h using Ir(III) complexes embedded into either polymer matrices^38^ or silica nanoparticles^39^. However, there is little prospect for Ir-based OPs, since they are critical raw materials due to low abundance, high production costs, and severe environmental impact (http://ec.europa.eu/growth/sectors/raw-materials/specific-interest/critical_en).

These limitations have brought biophosphors into focus, leading to Bio-HLEDs featuring performances close to those with Ir-based OPs^15,27–34^. Here, FP-phosphors stand out, since (i) the photoluminescence features of FPs fulfills all of the above requirements^27^, (ii) the protein backbone is an effective shield against ambient oxygen^40,41^, and (iii) FPs production can be carried out in a cheap and sustainable way using bacteria (please notice that lighting and laser applications do not require high purification levels).

The most critical aspect in the development of protein-phosphors is how to stabilize FPs out of aqueous solutions and physiological environments, preserving their bio-functionality over long periods of time. Although the idea of using FPs in lighting was proposed in 2000^42^, to date only three approaches have successfully realized FP-based Bio-HLEDs, namely (i) dry films prepared in a glass reservoir with a high concentration of FPs^32^, (ii) FPs encapsulated into MOFs^33^, and (iii) FP-polymer elastomeric coatings—i.e., a mixture of star-like hydrophilic polymer trimethylolpropane ethoxylate (TMPE) to stabilize the FPs and a linear polyethylene oxide polymer acting as a jellification agent^27–34^. The latter has provided the best performing devices with stabilities of <300 h^28,30^ and <5 min^31^ at low- and high-applied currents, respectively. The strong reduction in the device stability at high-applied currents is related to significant heat generation up to 60–70 °C upon continuous excitation of the FPs, whose origin is not well understood yet. Besides stability concerns, Bio-HLEDs also feature a limited efficiency due to the low transmittance of the elastomeric polymer matrix, reaching values <50 lm/W. Therefore, control of heat generation and photostabilization of FPs in polymer matrices with the appropriate optical features is instrumental for their use in lighting and laser applications.

In this context, this work presents a zero-thermal quenching FP-polymer phosphor, for which both, thermal- and photo-induced degradation processes and optical losses, are simultaneously circumvented embedding FPs into light-guiding and rigid hydrophobic host polymers. This biophosphor is inspired by previous observations about the role of green fluorescent proteins (GFPs) in (i) in vivo measurements of intracellular temperature combining temperature-induced molecular Brownian dynamics and polarized light excitation sources^43–47^ and (ii) body temperature control observed in living organisms like corals, which involves the mechanical change of the stiffness of the soft tissue where GFP clusters are embedded^48,49^. Like in single cells, vibrational and rotational motions of FPs might also be operative in elastomeric coatings due to the low viscosity of TMPE that partially replaces the water surrounding the FP’s surface. However, as in living organisms, this could be controlled adjusting the stiffness of the polymer network.

Based on these hypotheses, our approach to control heat generation in Bio-HLEDs involves the suppression of the FP motion using the stiffness of the polymer network surrounding the FPs. To this end, we developed a strategy to physically cross-link FPs shielded by the hydrophilic TMPE in a stiffer and hydrophobic host polymer like poly(methyl methacrylate) or PMMA. Success of this FP-polymer phosphor design is confirmed by the empirical relationship between the Young’s modulus of the coatings and the reduction of the temperature maxima to <30 °C in Bio-HLEDs operating at high-applied currents. This successfully preserved the bio-functionality of FPs in Bio-HLEDs enhancing orders of magnitude the device stability regardless of the applied current, reaching record stabilities beyond 150 days. Furthermore, this has been achieved while significantly increasing the efficiency of the device (ca. 130 lm/W) as a result of the high transmittance and light-guiding features of PMMA^50^.

## Results

### Fluorescent protein design

**Fig. 1:**
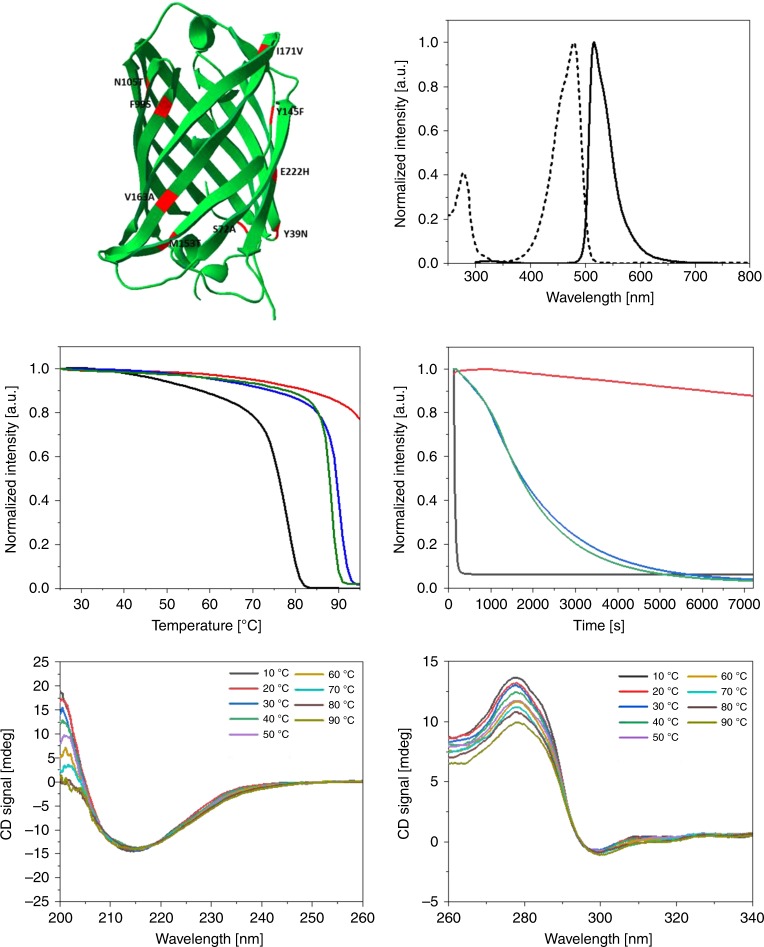
Characterization of GFP variants. Top: three-dimensional (3D) structure of the mutant eGFP-AA (left) (the 3D-model was made with Swiss PDB Viewer version 4.1 using the PDB file 2WUR as template) with the mutations E222H, Y39N, S72A, F99S, N105T, Y145F, M153T, V163A, and I171V highlighted in red, as well as absorption (dashed line) and emission (solid line) spectra of eGFP-AA in solution (right). Center: Photoluminescence (*λ*_exc_ = 488 nm) stability upon heating (left) and at 85 °C (right) of eGFP (black), eGFP-AA (red), eGFP-E222H (blue), and eGFP-FF (green). Bottom: CD spectra of eGFP-AA at different temperatures (see legend), showing the secondary structure (left) and the near-UV spectra reporting on the tertiary structure (right).

Previous studies in Bio-HLEDs have focused on classical FPs like enhanced GFP (eGFP), mCherry, and R-phycoerythrin, which are not well suited due to their propensity to exhibit photobleaching under irradiation, pH, and thermal stress scenarios. In light of these limitations, we have resorted to design an eGFP variant (eGFP-AA) implementing nine point-mutations in the structure of eGFP merging those previously used in the eGFP mutants eGFP-E222H^51^ and eGFP-FF^52^ (Fig. 1 and Supplementary Figs. 1 and 2, and Methods section for more details). The rationale behind is twofold. On one hand, the eGFP-E222H bears an exchange of the glutamic acid residue at position 222 by a histidine residue, eliminating the irreversible decarboxylation upon irradiation. On the other hand, eGFP-FF contains eight point-mutations—i.e., Y39N, S72A, F99S, N105T, Y145F, M153T, V163A, and I171V compared to the eGFP—that increase thermal tolerance and endow the mutant with fast-(re)folding characteristics enhancing the protein production (Supplementary Fig. 2).

The eGFP-AA shows similar photoluminescence features in aqueous solution to those of eGFP, eGFP-E222H, and eGFP-FF. In detail, the emission spectrum (*λ*_exc_ = 285 nm; Fig. 1 and Supplementary Fig. 3) consists of two emission bands centered at 326 and 516 nm corresponding to the fluorescence of Trp57 and the ionic form of the chromophore, respectively. This is associated with a *ϕ* of 85% and an average excited state lifetime (<*τ*>) value of 3.83 ns. Finally, both, the intensity ratio between these peaks (*I*_FP/Trp57_ ≈ 0.01), the lack of emission features originated from the neutral form of the chromophore (*λ*_em_ = 450 nm), and the prominent absorption centered at ca. 460 nm that relates to the ionic form of the chromophore (Fig. 1), are clear indications of an efficient folding of the protein^53,54^.

Besides maintaining the optimal folding and photoluminescence features of GFP-like proteins, the point-mutations implemented in eGFP-AA have also endowed this mutant with enhanced fluorescence thermostability and thermal melting temperature (*T*_m_), making it a more robust color down-converting material for lighting applications. Specifically, Fig. 1 shows that the *T*_m_ of eGFP-AA is >95 °C compared to 76, 90, and 88 °C for eGFP, eGFP-E222H, and eGFP-FF, respectively. eGFP-AA exhibits, in addition, an emission intensity decrease of 12.5% after 2 h at 85 °C, whereas the eGFP, eGFP-E222H, and eGFP-FF emission intensities decreased to 100, 96.1, and 96.7% of the initial intensity, respectively. To get insight into the structural stability of the β-barrel, circular dichroism (CD) assays were carried out (Fig. 1 and Supplementary Fig. 4). eGFP-AA demonstrated a high structural stability at high temperatures, showing negligible changes in the secondary structure and only a slight perturbation of the tertiary structure, as indicated by the change on the CD signal at 280 nm, which reports mostly on the local environment of the Trp57. These results suggest an increase in the β-barrel conformational flexibility with the raise in temperature from 10 to 90 °C. In addition, it is well-established that a drastic change in the protein structure in GFP proteins is always associated with the loss of both absorption and fluorescence features^55,56^, of the chromophore, which was not observed under any of the conditions tested.

### Polymer matrix design

**Fig. 2:**
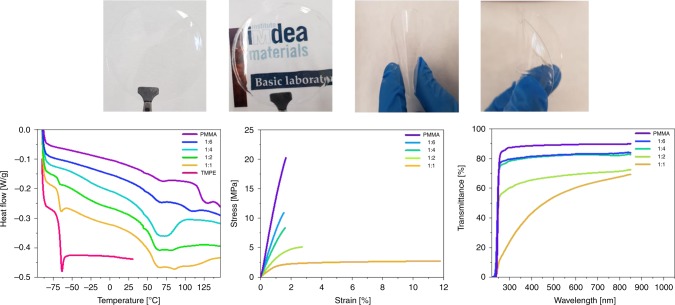
Polymer composite characterization. Top: Pictures of transparent and flexible 1:6 films. Bottom: DSC (left), tensile test (center), and transmittance (right) of films prepared with different mass ratios of TMPE:PMMA (see legend).

The key aspect to optimize the mechanical and optical features of the polymer network is the control of both, the viscosity of the gels and the blend nature of the thin-films, upon mixing the hydrophilic TMPE and hydrophobic PMMA polymers. Despite TMPE being a fluid, PMMA does not dissolve in this polymer even at high temperatures and high-speed stirring conditions. Thus, highly concentrated solutions of PMMA in acetonitrile were added to a certain amount of TMPE, achieving homogenous (to the naked eye) gels with mass ratios of 1:1, 1:2, 1:4, and 1:6 wt. of TMPE:PMMA. These gels were doctor-bladed onto a glass substrate and dried under vacuum (see Methods for details)^15,28,29^, realizing self-standing transparent films of ca. 150 μm (Fig. 2).

The blend nature of the thin-films was characterized using a comprehensive set of experimental techniques, namely (i) differential scanning calorimetry (DSC), (ii) X-ray diffraction (XRD), (iii) tensile test, (iv) scanning electron microscopy (SEM), and (v) steady-state UV–Vis transmittance. As shown in Fig. 2 and Supplementary Fig. 5, DSC assays identify three regions of glass transition temperatures (*T*_g_) going from −90 to 150 °C. The first region with a *T*_g_ value at around −60 °C corresponds to the presence of free TMPE zones given that the temperature onset appears at similar *T*_g_ value of pure TMPE. Thus, 1:1 and 1:2 thin-films, feature regions with pure TMPE segregated from the PMMA matrix. The second region with a *T*_g_ value of approximately −20 °C is consistent with a system exhibiting zones rich in TMPE, but interacting with PMMA, resulting in a *T*_g_ value shifted to higher temperatures than that of PMMA. This feature was observed for 1:1, 1:2, and 1:4 thin-films. This goes hand-in-hand with the observation of the *T*_g_ of PMMA plasticized by TMPE at ~80 °C in all of these samples. Altogether, this suggests that the blending is not fully homogeneous at these mass ratios. Indeed, SEM pictures confirmed the presence of two phases entwined in a co-continuous fashion for 1:1 films, while the others showed a continuous phase dominated by PMMA, in which the TMPE regions are well-distributed and become smaller upon reducing its amount (Supplementary Fig. 6). In contrast, 1:6 thin-films showed exclusively the thermal features characteristic of a single-phase polymer blend (please notice that the endothermic peak that appears concealing the *T*_g_ step relates to the physical aging of the PMMA chains. This is substracted with a MDSC as shown in Supplementary Fig. 5), whose morphology is, indeed, very similar to that of PMMA (Supplementary Fig. 6).

To further support these findings, tensile test measurements were carried out (Fig. 2). The first difference was observed in the elongation at break, where 1:1 films break at 12%, while the others feature similar values to those of PMMA—i.e., 3%. This is rationalized in terms of the blend morphology, since only 1:1 films presented a clear co-continuous phase that favors the ductile behavior of this blend. The others show a quite homogeneous morphology dominated by the PMMA and, in turn, they feature a similar brittle behavior. However, Young’s modulus increases from 173.7 to 303 MPa, to 577 MPa, to 783.4 MPa, and to 1371 MPa for 1:1, 1:2, 1:4, 1:6, and PMMA thin-films. The increase in Young’s modulus is expected, since both, DSC and SEM assays, show the presence of less phases and/or TMPE-PMMA interfaces going from 1:2 to 1:6 thin-films. This conclusion was also corroborated characterizing the degree of amorphization using XRD (Supplementary Fig. 5). The XRD spectra of the thin-films resemble those of PMMA films, showing a broad peak centered at 2*θ* = 15° for 1:1 and 1:2 films that gets narrower and is similar to that of pure PMMA (2*θ* = 13°) for 1:4 and 1:6 films. This indicates an optimum degree of compatibility and homogeneity in 1:6 films even if FPs (1 mg) are present (Supplementary Fig. 5). Finally, the homogeneous nature of the blended films is also revealed in the transmittance that increases going from 1:1 to 1:6 thin-films (Fig. 2). Indeed, a simple test to determine photon flux losses at different vision angles carried out with a blue LED (440 nm) shows a linear relationship with the transmittance of the films at any angle (Supplementary Fig. 5), further confirming the homogeneous nature of the blended films.

### FP-polymer bio-phosphors: preparation and characterization

**Fig. 3:**
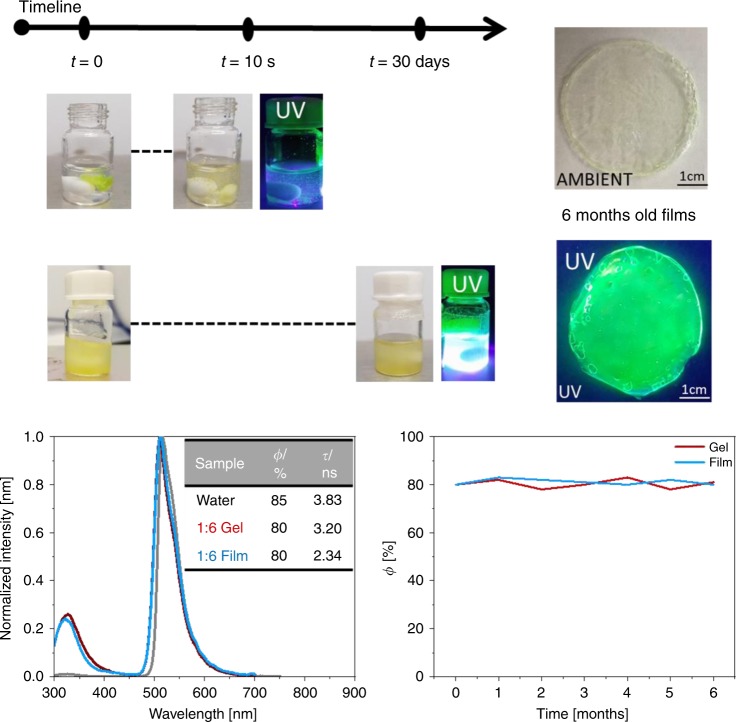
FP-polymer bio-phosphor characterization. Top: pictures at room light and under UV irradiation of mixtures of eGFP-AA in acetonitrile-based PMMA gels (top) and acetonitrile-based TMPE:PMMA (1:6) gels (bottom), highlighting their temporal changes. Pictures of 6 months-old eGFP-AA doped 1:6 films under room light and UV irradiation are shown at the right side. Bottom: Emission spectra (left) of 1:6 gels and 1:6 films with eGFP-AA upon excitation at 285 nm. The inset table gathers the corresponding *ϕ* and *τ*, while the time dependent behavior of *ϕ* under storage conditions is also shown (right).

As next step, we determined the stability of eGFP-AA embedded in the mixture of TMPE:PMMA in both acetonitrile-based gel and film forms. As a matter of fact, the direct mixture of FPs into a highly concentrated acetonitrile solution of PMMA leads to a quick denaturation of the proteins visible to the naked eye (Fig. 3). In this respect, several groups have immobilized bio-compounds on the surface, microchannels, vesicles, fibers, etc. using self-assembly and/or cross-linking with PMMA-based block copolymers, keeping a water environment to preserve the bio-functionality^57–59^. These approaches rely, however, on chemical modifications of PMMA and chemical reactions with the bio-compounds, risking the stability and optical features of the final materials. To circumvent these issues, we have shown that the hydrophilic TMPE polymer significantly stabilizes FPs in gel and elastomeric forms with a high content of water under storage and device operating conditions^15,27,30,31^. Now, we state that this approach can also be applied to stabilize FPs in very dry media, shielding the surrounding of the FP structure against the toxic polymer solution environment for long periods of time (Fig. 3). This is confirmed by the fact that the mixture of eGFP-AA into 1:6 TMPE:PMMA gels shows an exceptional stability over months, as highlighted by the neglectable change in the photoluminescence features (Fig. 3).

Furthermore, films with eGFP-AA obtained from these gels show photoluminescence features fairly similar to those in water (Fig. 3). In detail, the emission spectra are slightly red-shifted (*λ*_em_ = 509 nm) in concert with reduced *ϕ* (80%) and <*τ*> (2.34 ns) values. These changes are attributed to small structural arrangements of the protein backbone that slightly affect the polarity at the chromophore cavity, since the *I*_FP/Trp57_ value does not increase and the emission features of the neutral form of the chromophore are not observed^53,54^. Finally, we noted a remarkable stability under different scenarios (see Methods for details and Supplementary Fig. 7). While the 1:6 films are stable over months under storage at ambient conditions (Fig. 3), a <10% loss in *ϕ* was noted at either constant UV irradiation (365 nm) or 50 °C in air after weeks (Supplementary Fig. 7). Likewise, *ϕ* also remained constant after immersing the films in water (Supplementary Fig. 7).

### Bio-HLEDs: preparation and characterization

**Fig. 4:**
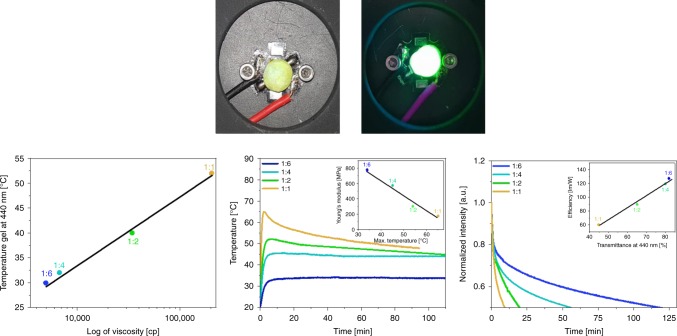
Bio-HLED characterization. Top: pictures of Bio-HLEDs at work. Bottom: linear relationship (*R*^2^ = 0.99 for linear fitting, solid black line) between heat generation and viscosity (left) of eGFP-AA doped TMPE:PMMA gels with different mass ratios. Increase in temperature (center) and emission intensity decay (right) over time in Bio-HLEDs operating at 200 mA for biophosphors consisting of eGFP-AA doped TMPE:PMMA filters with different mass ratios (see legend). The inset shows the linear relationship (*R*^2^ > 0.98 for linear fittings, solid black lines) between (i) maximum temperature of the devices and Young’s modulus of the biophosphors (center) and (ii) device efficiency and transmittance of the filters (right).

Having stabilized the eGFP-AA into a mixture of TMPE:PMMA in both gel and film forms, we turned to validate our initial hypothesis that FP motion triggered by constant irradiation is responsible for the heat generation in HLEDs. Following works on intracellular thermometry in cells^43–47^ and living organism like corals^48,49^, where it is shown that the viscosity of the media is key to control the heat generation, we first analyzed the increase in temperature of eGFP-AA gels with different TMPE:PMMA mass ratios (see SI for more details). The maximum temperature reduced from 52 °C to 40 °C, to 32 °C, and to 30 °C upon increasing the viscosity of the gels for 1:1, 1:2, 1:4, and 1:6 mass ratios, respectively (Fig. 4). Interestingly, the same trend was noted in Bio-HLEDs, in which these color filters were directly placed onto a high-power blue LED chip (200 mA) (Fig. 4); see Methods for details. All the Bio-HLEDs showed a good blue-to-green energy conversion with a dominant emission band centered at around 515 nm (Supplementary Fig. 8), and a prominent rise in temperature, reaching its maximum value after 8–10 min (Fig. 4). Notice that neither the emitting chip nor the FP-free color filter experience an increase of the temperature beyond 32 °C under these operating conditions. In line with the heat generation in the gels, the maximum temperature under device operation decreases from 65 °C to 52 °C, to 46 °C, and to 35 °C for 1:1, 1:2, 1:4, and 1:6 filters, respectively. This goes hand-in-hand with the increase of the stiffness (Young’s modulus) of the films (Fig. 4). Noteworthy, these trends must be considered as phenomenological relationships that need to be supported by a theoretical model, in which the dynamic changes of the hydrodynamic volume of the FPs-polymers, the free volume effects in the TMPE regions, friction forces, and heat dissipation may be taken into consideration. Nevertheless, the strong reduction in temperature leads to an exponential increase in the device lifetime—i.e., time needed to reach half of the initial emission (*L*_50_)—from 8 min to 2 h for Bio-HLEDs with 1:1 and 1:6 filters (Fig. 4 and Supplementary Fig. 8). In addition, the luminous efficiency shows a twofold enhancement going from 1:1 (60 lm/W) to 1:6 (127 lm/W) nicely following the increase in the transmittance values of the filters (Fig. 4). This is in line with the reduced optical losses related to an enhancement of the light out-coupling factor with the transmittance^60^. Striking enough, Bio-HLEDs with 1:6 filters significantly outperform the reference devices with eGFP-AA based on elastomeric filters^15,27,30^, reaching efficiencies of 38.7 lm/W and *L*_50_ of 5.6 min (Supplementary Fig. 8). Note, also that previous Bio-HLEDs based on elastomeric filters with eGFP featured efficiencies of 27 lm/W and *L*_50_ of 2 min at the same driving conditions^31^. This highlights the prospect of the mutant regardless of the chosen polymer matrices.

Hence, we conclude that the origin of the heat generation in FP-polymer filters is related to the motion of the FPs upon continuous excitation. This effect can be controlled by the stiffness of the polymer network surrounding the FPs acting as an effective straitjacket that prevents heat generation. Note that changes in the thermal conductivity among the filters are neglectable, since TMPE and PMMA show values of 0.238 and 0.250 W/mK, respectively. In addition, the increase in transmittance is also a great asset, leading to highly efficient devices compared to those of the prior-art.

**Fig. 5:**
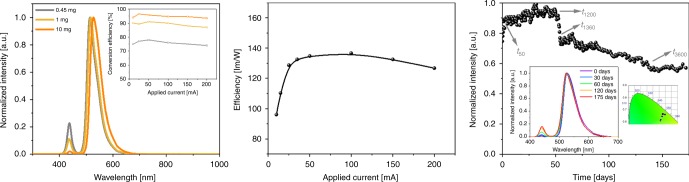
Bio-HLED stability studies. Left: emission of Bio-HLEDs with biophosphors containing different amounts of eGFP-AA at a driving current of 30 mA. The inset graphs display the changes in the conversion efficiency values upon increasing the applied current. Center: changes in the device efficiency upon increasing the applied current. Right: changes in the intensity of the biophosphor emission over time in Bio-HLEDs driven at 30 mA. The inset shows the changes in the overall electroluminescence spectra (left) and its *x*/*y* CIE color coordinates (right) of Bio-HLEDs over time.

Despite the significant enhancement of the device performance using 1:6 filters, both device stability and blue-to-green conversion values can be further improved increasing the amount of the eGFP-AA content going from 0.45 mg to 1 mg, and to 10 mg (Fig. 5 and Supplementary Fig. 9). Upon increasing the protein amount, the emission band broadens and red-shifts, showing reduced *ϕ* and increased <*τ*> values (Supplementary Fig. 9). In addition, the *I*_FP/Trp57_ value is further reduced to ~0.05, while the emission features of the neutral form of the chromophore are not present. All these photophysical features point to a protein aggregation effect prior to the encapsulation into the PMMA polymer.

As expected, the conversion efficiency—i.e., LED emission ratio area between FP- coating/LED and coating/LED—increases with the amount of eGFP-AA present in the 1:6 color filters, reaching a value >90% for Bio-HLEDs with 10 mg eGFP-AA filters at applied currents of 200 mA (Fig. 5). Much more relevant, devices fabricated with these color filters do not show a significant increase in temperature (32–34 °C even after 2 h) at high-applied currents (200 mA). Finally, a maximum efficiency of 134 lm/W was reached at applied currents of 30 mA, showing the typical decay curve due to the internal loss of the emitting chip at higher applied currents (Fig. 5).

Using this driving condition (30 mA), the stability of the Bio-HLEDs was monitored over time (Fig. 5). Despite the slight increase in the coating temperature that might lead to thermal quenching, the intensity of the eGFP-AA emission band increases during the first 50 h (*t*_50_). This goes hand-in-hand with an increase in *ϕ* up to ca. 75% that points out to a temperature-induced disaggregation of the FPs in the coating. An increase in the *I*_FP/Trp57_ (0.07) is also noted, while the emission features at 450 nm are not observed, discarding changes in the state of the chromophore (Supplementary Fig. 10). Beyond *t*_50_, both intensity and emission band shape hold constant until 1200 h, when the intensity quickly reduces reaching a new plateau at 1360 h (*t*_1360_). Here, steady-state photoluminescence assays show that the maximum wavelength of emission is slightly blue-shifted (5 nm), while the emission features of the neutral form of the chromophore (*λ*_em_ = 450 nm) evolved along with an increase of *I*_FP/Trp57_ (0.15) and a reduction of *ϕ* (60%) (Supplementary Fig. 10). This resembles the photo-induced deactivation mechanism recently reported in Bio-HLEDs with elastomeric filters operating without temperature stress^27,31^. In short, this consists of several dehydration or H^+^-transfer steps, in which the nature of the chromophore changes from the highly emissive ionic form to its poorly emissive neutral form. This is promoted by small conformational changes of the Trp57 surroundings located at the end of the inner helix and at the beginning of an interconnection loop that closes the β-barrel. These changes are monitored with the increase of the *I*_FP/Trp57_^53,54^ that is twofold compared to fresh samples. These small structural distortions facilitate the inside/outside H^+^ transfer of the β-barrel, changing the local environment of the chromophore as the emission features of the neutral form appeared. However, this photo-induced deactivation process is very slow, showing different steady regimes. Indeed, the emission intensity of the Bio-HLED slowly and linearly decreases until it reaches another plateau starting at around 3600 h (*t*_3600_) operating time. This point represents a record *L*_50_ of >150 days. Over this time, the degradation mechanism evolves due to i) the increase in the *I*_FP/Trp57_ value (0.29) and in the intensity of the neutral chromophore emission and ii) the decrease of *ϕ* values down to ca. 40%. Compared to elastomeric filters used in Bio-HLEDs^27,31^, the use of rigid filters significantly slows down this photo-induced degradation process restraining the movement of the proteins and limiting the amount of water surrounding the protein backbone. This leads to a significant enhancement of the device stability compared to elastomeric filters from minutes to hours and from hundreds of hours to thousands of hours operating under high (200 mA) and low (30 mA) powers, respectively.

## Discussion

We have demonstrated that the origin of the temperature rise, leading to a poor Bio-HLED stability of a few minutes is caused by the FP motion upon excitation. This effect can be circumvented with the presented biophosphor concept, in which the FPs are embedded into a stiffer polymer surrounding like PMMA using the hydrophilic TMPE polymer as a shielding interface. This preserves the bio-functionality of the FPs both in gels and films over months under storage conditions and weeks under UV irradiation, temperature, and moisture stress scenarios. The optimized biophosphors feature a single-phase structure with high transmittance and Young’s modulus values that lead to highly efficient and stable Bio-HLEDs regardless of the amount of FPs and device operating conditions. These conclusions are supported by (i) the enhancement of the device stability and efficiency compared to those of Bio-HLEDs with elastomeric filters, and (ii) the empirical relationships between the stiffness of the biophosphors and the maximum temperature reached under device operation, as well as the transmittance of the filters and the device efficiency. As the most remarkable result, long-living (>150 days) and highly efficient (~130 lm/W) Bio-HLEDs were realized. This strongly contrasts with the prior-art in Bio-HLEDs based on FPs—i.e., average <300 h and <50 lm/W^27–34^, those based on bio-matrices—i.e., average <200 h and <10 lm/W^15,22–26^, other HLEDs based on small molecules—i.e., <1000 h and <150 lm/W^16–18^ and iridium(III) complexes—i.e., <1000 h and <100 lm/W^38,39^. Hence, this work opens a way to stabilize FPs in common polymer matrices for optics, providing a significant advance in the emerging Bio-HLED concept.

## Methods

### Cloning of gene constructs

All mutated eGFP constructs were ordered as synthetic gene strings (GeneArt, Thermo Fisher Scientific) with BamHI (GGATCC) and SalI (GTCGAC) restriction sites for subsequent cloning. The synthetic gene strings were diluted according to manufacturer’s instructions. The sequence of eGFP wild type was amplified from the Gateway vector pK7FWG2.0^61^ using polymerase chain reaction (PCR) with gene-specific primers. High-Fidelity Phusion DNA-polymerase (Thermo Fisher Scientific) was used to perform the PCR amplification. All constructs were first ligated in the cloning vector pCRBlunt (Invitrogen) via T4 DNA ligase reaction (Thermo Fisher Scientific) and transformed in *Escherichia coli* XL1Blue cells to multiply the plasmids. Afterwards, the plasmids containing the eGFP constructs were isolated by plasmid isolation, digested via BamHI/SalI restriction digestion and separated on agarose gels (restriction enzymes from Thermo Fisher Scientific). The digested genes of interest were extracted and purified via gel Extraction Kit according to manufacturer’s instructions (Gel Extraction Kit, Qiagen). The purified fragments were ligated into the *E. coli* expression vector pQE-9 (Qiagen) via restriction sites using T4 DNA ligase (Thermo Fisher Scientific). The pQE-9 plasmid is IPTG-inducible and contains a 6xHis affinity tag that is fused to the N-terminus of the protein during expression. The plasmids were transformed in chemically competent *E. coli* XL1Blue cells for sequence verification using Sanger Sequencing (GATC Biotech).

### Recombinant protein expression

The pQE-9 plasmids were transformed in the chemically competent bacterial expression strain *E. coli* M15 [pREP4] (Qiagen) and plated on Luria-Bertani (LB) agar plates containing 200 μg/mL ampicillin and 25 μg/mL kanamycin. Single colonies were picked for inoculating an overnight culture in liquid LB with antibiotics (10 g NaCl, 10g Bacto-tryptone, 5 g yeast extract per 1 l; 200 µg/mL ampicillin and 25 µg/mL kanamycin). The culture was incubated overnight at 37 °C. Appropriate amounts of the overnight culture were used to inoculate a 1 l expression culture to an optical density (OD_600_) of 0.2. The expression cultures were cultivated at 37 °C and shaken at 180–200 rpm until an OD_600_ of 0.5 was reached. Recombinant protein expression was induced by addition of 1 mM IPTG (Roth) for 4 h. After 4 h, cells were harvested by centrifugation at 5000 × *g* for 20 min at 4 °C. Cell pellets were frozen at −20 °C for storage. To monitor the expression of recombinant proteins, samples were taken before induction (0 h) and then every hour until harvest (1 h–4 h) to perform expression kinetics. Samples were adjusted to the same OD_600_ using 4xLaemmli buffer (200 mM Tris-HCl, pH 6.8, 18% β-mercaptoethanol, 40% glycerol, 0.01% bromophenol blue, and 8% SDS) prior to boiling at 95 °C for 10 min. Boiled samples were separated on a SDS-Page (12% Bis-Tris gel) and later stained with Coomassie Brilliant Blue.

### Recombinant protein purification

Protein purification of recombinant expressed 6xHis tagged proteins was performed via nickel-nitrilotriacetic acid agarose (Ni-NTA, Qiagen) affinity chromatography under native conditions (non-denaturing). Harvested cell pellets were thawed on ice and resuspended in lysis buffer containing 1 mM Pefabloc (Roth) and cOmplete Ultra tablets, EDTA-free protease inhibitor (Merck) to minimize protein degradation. Sonification steps were performed on ice for efficient cell lysis (10 s bursts with cooling on ice after each burst). Soluble and insoluble components of cell lysate were separated by centrifugation (10,000 × *g*, 30 min, 4 °C). For further purification, the supernatant fraction containing all soluble proteins was used and applied to Ni-NTA agarose resin packed in polypropylene columns (5 mL, Qiagen). The following purification steps, including binding, washing, and elution steps were performed according to the manufacturer’s instructions (QiaExpressionist, Qiagen 2001). The following buffers were used: lysis buffer (50 mM NaH_2_PO_4_ pH 8.0; 300 mM NaCl; 10 mM imidazole); washing buffer (50 mM NaH_2_PO_4_ pH 8.0; 300 mM NaCl; 20 mM imidazole); elution buffer (50 mM NaH_2_PO_4_ pH 8.0; 300 mM NaCl; 250 mM imidazole). Subsequently, the proteins were dialyzed against PBS-buffer (136 mM NaCl, 2.7 mM KCl, 8 mM Na_2_HPO_4_, and 1.8 mM KH_2_PO_4_, pH 7.4) to remove imidazole using dialysis tubing with a molecular weight cutoff of 12–14 kDa (SERVAPOR dialysis tubing, SERVA). If needed, the dialyzed protein was concentrated using Amicon Ultra-4 centrifugal filter units (MWCO 10 K, Merck). Protein concentration of recombinant proteins was determined at 280 nm with a NanoDrop spectrophotometer ND-1000 (Peqlab).

### Sequences of eGFP wild type and mutants

#### eGFP wild type

GGATCCGTGAGCAAGGGCGAGGAGCTGTTCACCGGGGTGGTGCCCATCCTGGTCGAGCTGGACGGCGACGTAAACGGCCACAAGTTCAGCGTGTCCGGCGAGGGCGAGGGCGATGCCACCTACGGCAAGCTGACCCTGAAGTTCATCTGCACCACCGGCAAGCTGCCCGTGCCCTGGCCCACCCTCGTGACCACCCTGACCTACGGCGTGCAGTGCTTCAGCCGCTACCCCGACCACATGAAGCAGCACGACTTCTTCAAGTCCGCCATGCCCGAAGGCTACGTCCAGGAGCGCACCATCTTCTTCAAGGACGACGGCAACTACAAGACCCGCGCCGAGGTGAAGTTCGAGGGCGACACCCTGGTGAACCGCATCGAGCTGAAGGGCATCGACTTCAAGGAGGACGGCAACATCCTGGGGCACAAGCTGGAGTACAACTACAACAGCCACAACGTCTATATCATGGCCGACAAGCAGAAGAACGGCATCAAGGTGAACTTCAAGATCCGCCACAACATCGAGGACGGCAGCGTGCAGCTCGCCGACCACTACCAGCAGAACACCCCCATCGGCGACGGCCCCGTGCTGCTGCCCGACAACCACTACCTGAGCACCCAGTCCGCCCTGAGCAAAGACCCCAACGAGAAGCGCGATCACATGGTCCTGCTGGAGTTCGTGACCGCCGCCGGGATCACTCTCGGCATGGACGAGCTGTACAAGTAA

G S V S K G E E L F T G V V P I L V E L D G D V N G H K F S V S G E G E G D A T Y G K L T L K F I C T T G K L P V P W P T L V T T L T Y G V Q C F S R Y P D H M K Q H D F F K S A M P E G Y V Q E R T I F F K D D G N Y K T R A E V K F E G D T L V N R I E L K G I D F K E D G N I L G H K L E Y N Y N S H N V Y I M A D K Q K N G I K V N F K I R H N I E D G S V Q L A D H Y Q Q N T P I G D G P V L L P D N H Y L S T Q S A L S K D P N E K R D H M V L L E F V T A A G I T L G M D E L Y K *

#### eGFP-AA (double mutant)

GGATCCGTGAGCAAGGGCGAGGAGCTGTTCACCGGGGTGGTGCCCATCCTGGTCGAGCTGGACGGCGACGTAAACGGCCACAAGTTCAGCGTGTCCGGCGAGGGCGAGGGCGATGCCACCAACGGCAAGCTGACCCTGAAGTTCATCTGCACCACCGGCAAGCTGCCCGTGCCCTGGCCCACCCTCGTGACCACCCTGACCTACGGCGTGCAGTGCTTCGCGCGCTACCCCGACCACATGAAGCAGCACGACTTCTTCAAGTCCGCCATGCCCGAAGGCTACGTCCAGGAGCGCACCATCAGCTTCAAGGACGACGGCACCTACAAGACCCGCGCCGAGGTGAAGTTCGAGGGCGACACCCTGGTGAACCGCATCGAGCTGAAGGGCATCGACTTCAAGGAGGACGGCAACATCCTGGGGCACAAGCTGGAGTACAACTTTAACAGCCACAACGTCTATATCACCGCCGACAAGCAGAAGAACGGCATCAAGGCGAACTTCAAGATCCGCCACAACGTGGAGGACGGCAGCGTGCAGCTCGCCGACCACTACCAGCAGAACACCCCCATCGGCGACGGCCCCGTGCTGCTGCCCGACAACCACTACCTGAGCACCCAGTCCGCCCTGAGCAAAGACCCCAACGAGAAGCGCGATCACATGGTCCTGCTGCATTTCGTGACCGCCGCCGGGATCACTCTCGGCATGGACGAGCTGTACAAGTAA

G S V S K G E E L F T G V V P I L V E L D G D V N G H K F S V S G E G E G D A T **N** G K L T L K F I C T T G K L P V P W P T L V T T L T Y G V Q C F **A** R Y P D H M K Q H D F F K S A M P E G Y V Q E R T I **S** F K D D G **T** Y K T R A E V K F E G D T L V N R I E L K G I D F K E D G N I L G H K L E Y N **F** N S H N V Y I T A D K Q K N G I K **A** N F K I R H N **V** E D G S V Q L A D H Y Q Q N T P I G D G P V L L P D N H Y L S T Q S A L S K D P N E K R D H M V L L **H** F V T A A G I T L G M D E L Y K *

#### eGFP-FF^52^

GGATCCGTGAGCAAGGGCGAGGAGCTGTTCACCGGGGTGGTGCCCATCCTGGTCGAGCTGGACGGCGACGTAAACGGCCACAAGTTCAGCGTGTCCGGCGAGGGCGAGGGCGATGCCACCAACGGCAAGCTGACCCTGAAGTTCATCTGCACCACCGGCAAGCTGCCCGTGCCCTGGCCCACCCTCGTGACCACCCTGACCTACGGCGTGCAGTGCTTCGCGCGCTACCCCGACCACATGAAGCAGCACGACTTCTTCAAGTCCGCCATGCCCGAAGGCTACGTCCAGGAGCGCACCATCAGCTTCAAGGACGACGGCACCTACAAGACCCGCGCCGAGGTGAAGTTCGAGGGCGACACCCTGGTGAACCGCATCGAGCTGAAGGGCATCGACTTCAAGGAGGACGGCAACATCCTGGGGCACAAGCTGGAGTACAACTTTAACAGCCACAACGTCTATATCACCGCCGACAAGCAGAAGAACGGCATCAAGGCGAACTTCAAGATCCGCCACAACGTGGAGGACGGCAGCGTGCAGCTCGCCGACCACTACCAGCAGAACACCCCCATCGGCGACGGCCCCGTGCTGCTGCCCGACAACCACTACCTGAGCACCCAGTCCGCCCTGAGCAAAGACCCCAACGAGAAGCGCGATCACATGGTCCTGCTGGAGTTCGTGACCGCCGCCGGGATCACTCTCGGCATGGACGAGCTGTACAAGTAA

G S V S K G E E L F T G V V P I L V E L D G D V N G H K F S V S G E G E G D A T **N** G K L T L K F I C T T G K L P V P W P T L V T T L T Y G V Q C F **A** R Y P D H M K Q H D F F K S A M P E G Y V Q E R T I **S** F K D D G **T** Y K T R A E V K F E G D T L V N R I E L K G I D F K E D G N I L G H K L E Y N **F** N S H N V Y I **T** A D K Q K N G I K **A** N F K I R H N **V** E D G S V Q L A D H Y Q Q N T P I G D G P V L L P D N H Y L S T Q S A L S K D P N E K R D H M V L L E F V T A A G I T L G M D E L Y K *

#### eGFP-E222H^51^

GGATCCGTGAGCAAGGGCGAGGAGCTGTTCACCGGGGTGGTGCCCATCCTGGTCGAGCTGGACGGCGACGTAAACGGCCACAAGTTCAGCGTGTCCGGCGAGGGCGAGGGCGATGCCACCTACGGCAAGCTGACCCTGAAGTTCATCTGCACCACCGGCAAGCTGCCCGTGCCCTGGCCCACCCTCGTGACCACCCTGACCTACGGCGTGCAGTGCTTCAGCCGCTACCCCGACCACATGAAGCAGCACGACTTCTTCAAGTCCGCCATGCCCGAAGGCTACGTCCAGGAGCGCACCATCTTCTTCAAGGACGACGGCAACTACAAGACCCGCGCCGAGGTGAAGTTCGAGGGCGACACCCTGGTGAACCGCATCGAGCTGAAGGGCATCGACTTCAAGGAGGACGGCAACATCCTGGGGCACAAGCTGGAGTACAACTACAACAGCCACAACGTCTATATCATGGCCGACAAGCAGAAGAACGGCATCAAGGTGAACTTCAAGATCCGCCACAACATCGAGGACGGCAGCGTGCAGCTCGCCGACCACTACCAGCAGAACACCCCCATCGGCGACGGCCCCGTGCTGCTGCCCGACAACCACTACCTGAGCACCCAGTCCGCCCTGAGCAAAGACCCCAACGAGAAGCGCGATCACATGGTCCTGCTGCATTTCGTGACCGCCGCCGGGATCACTCTCGGCATGGACGAGCTGTACAAGTAA

G S V S K G E E L F T G V V P I L V E L D G D V N G H K F S V S G E G E G D A T Y G K L T L K F I C T T G K L P V P W P T L V T T L T Y G V Q C F S R Y P D H M K Q H D F F K S A M P E G Y V Q E R T I F F K D D G N Y K T R A E V K F E G D T L V N R I E L K G I D F K E D G N I L G H K L E Y N Y N S H N V Y I M A D K Q K N G I K V N F K I R H N I E D G S V Q L A D H Y Q Q N T P I G D G P V L L P D N H Y L S T Q S A L S K D P N E K R D H M V L L **H** F V T A A G I T L G M D E L Y K *

Mutated amino acids compared to wild type eGFP are highlighted in bold.

Gene-specific primers for the amplification of eGFP wild type from pK7FWG2,0^61^.

eGFP_FW: 5′ GGATCCGTGAGCAAGGGCGAGGAGCTGTTC 3′eGFP_RV: 5′ GTCGACTTACTTGTACAGCTCGTCCATG 3′.

### Circular dichroism

The protein secondary and tertiary structure was examined by circular dichroism (CD) using a Jasco J-815 spectrometer. CD spectra to monitor the secondary structure were acquired at 10 μM protein concentration in 150 mM NaCl, 50 mM Tris/HCl pH 8.0 using 0.1 cm pathlength cuvette, a band-width of 1 nm at 1 nm increments, and 10 s average time over a wavelength range of 200 to 260 nm. The tertiary structure was monitored at 40 µM protein concentration under the same buffer conditions in the near-UV region from 260 to 340 nm. The protein stability was evaluated measuring the thermal denaturation curves and monitored by following the CD signal at 215 and 277 nm as a function of temperature from 10 to 90 °C.

### Fluorescence thermal denaturation

Fluorescence thermal denaturation experiments were carried out using a StepOne Real-time PCR system (Applied Biosystems). Data were acquired exciting at 488 nm and measuring the fluorescence emission at 509 nm using a temperature ramp of 0.5 °C/min from 25 to 95 °C. The thermal stability at a constant temperature was determined using the same equipment by fixing the temperature at 85 °C and measuring the fluorescence emission intensity for 120 min.

### Preparation and characterization of the FP-polymer phosphors and devices

All chemicals were purchased from Sigma Aldrich and used without further purification. The branched (i.e., trimethylolpropane ethoxylate or TMPE) with Mn. of 450 mol. wt. and poly (methyl methacrylate) (PMMA) with Mn. of 3.5 × 10^5 ^mol. wt were used. Films over 100 microns of thickness were prepared via drop casting. TMPE was weighed first and an 83.3 mg/mL solution of PMMA in acetonitrile was added and vigorously stirred over a couple of minutes, then the mixture was poured into a petri dish and dried under vacuum conditions for ~3 h 30 min using a vacuum ramp varying from 200 to 3 mbar. The color filters for the devices were produced in a similar procedure as the films mentioned above. TMPE was weighed first and mixed with FP. An 83.3 mg/mL solution of PMMA in acetonitrile was added and vigorously stirred over a couple of minutes, then the mixture was concentrated under vacuum until it exhibited a rubber-like appearance. The rubber-like mixture was then molded into the shape of interest (dome with 6 mm diameter and 1 mm thickness) and dried under vacuum conditions for ~3 h 30 min using a vacuum ramp varying from 200 to 3 mbar.

The photophysical studies were carried out using a FS5 Spectrofluorometer (Edinburgh Instruments) with the SC-10 module for solid samples, the SC-30 Integrating Sphere to determine *ϕ*, and the 375 nm time-correlated single photo-counting or TCSPC (64.3 ps pulse width) module to determine *τ*. The data was then adjusted to a bi-exponential decay fit using Origin 8b. To calculate the average lifetime for each FP-coating, the following equation was used $< \tau > \; = \frac{{{\int_0^x} t \,{\sum} {a_i\,exp} \big( { - \frac{t}{{\tau _i}}} \big)dt}}{{{\int_0^x} {\sum} {a_i\,exp} \big( { - \frac{t}{{\tau _i}}} \big)dt}} = \frac{{{\sum} {a_i\tau _i^2} }}{{{\sum} {a_i\tau _i} }}$; ref. ^62^ where *a*_*i*_ (λ) is the amplitude fractions and *τ*_*i*_ are the lifetimes. The measurements were performed at room temperature. UV–Vis spectroscopy was realized using the absorption module and setting up the device to scan speed medium, accumulation 0.1, alit width 2.0 nm and sampling intervals of 1.0 nm. The temperature increase of the gels was studied by placing them in a glass reservoir irradiated with a blue laser (450 nm), while recording the temperature of the gel with a thermographic camera (T430sc; FLIR).

Blending of different ratio films was tested and characterized with various techniques. Mechanical characterization was carried out with a Q800 DMA from TA Instruments, using a tension clamp. The assay was done with a rectangular (15 × 4.9 × 0.1mm) shape sample and tested in a ramp force of 1 N/m. Calorimetric essays for characterization (DSC) were carried out using a Q200 DSC from TA Instruments.

The degree of amorphization was studied using XRD performed by PANalytical Empyrean X-Ray diffractometer equipped with a PIXcel1D detector with Cu Kα radiation at 45 kV and 40 mA. Divergence optics with a beam mask of 10 mm and a fixed divergence slit of 1/16° were applied using a *θ*/2*θ* scan from 4 to 35° with 2*θ* steps of 0.039° and 400 s of acquisition time per step. The detector was used at one-dimensional scanning line mode.

The morphology of the polymer blends was investigated by scanning electron microscopy (SEM) using a FEI Helios Nanolab 600i–SEM operating at an accelerating voltage of 3 kV. The above films were cooled down and were broken. Finally, all samples were first sputtered with 30 nm of Au performed by Q150T ES from Quorum technologies^63^.

The stability test under stress scenarios include: (i) stability in a water reservoir (type 2 and pH = 7.1), (ii) UV irradiation (365 nm; 8W), and (iii) thermostability at 50 °C. For moisture studies, the sample was deposited in a water reservoir, while the temperature of the sample under both the UV irradiation (25–27 °C) and thermal (50 °C) stresses was monitored with a thermographic camera T430sc (FLIR). The PLQY changes of the samples were monitored over time (fresh, 1, 3, 5, 24, 48 h, etc.) using a FS5 Spectrofluorometer (Edinburgh Instruments) with the SC-10 module for solid samples and the SC-30 Integrating Sphere to determine *ϕ*.

The biophosphors were directly placed onto the 440 nm LED (Winger Electronics). The Bio-HLEDs were characterized using a Keithley 2400 as a current source, while the changes in the electroluminescence spectrum were monitored using an AVS-DESKTOP-USB2 (Avantes) in conjunction with a calibrated integrating sphere Avasphere 30-Irrad, while the changes in the FP-coating temperature were monitored using a thermographic camera T430sc (FLIR) coupled to the measuring system.

## Supplementary information

Supplementary Information

## Data Availability

The authors declare that the data supporting the findings of this study are available within the paper and its supplementary information files.
