# Supplementary material for: Long-living and highly efficient bio-hybrid light-emitting diodes with zero-thermal-quenching biophosphors
Source: Nat Commun. 2020 Feb 13;11:879. doi: 10.1038/s41467-020-14559-8 (PMC7018753; doi:10.1038/s41467-020-14559-8)
Supplement: Supplementary file 1 — Supplementary Information [file 41467_2020_14559_MOESM1_ESM.pdf]

Supplementary Information of

Long-living and Highly Efficient Bio-hybrid Light-emitting Diodes with Zero-thermal-quenching Biophosphors

By A. Espasa, M. Lang, C. F. Aguiño, D. Sanchez-deAlcazar, J. P. Fernández-Blázquez, U. Sonnewald, A. L. Cortajarena, P. B. Coto and R. D. Costa

1. Supplementary Figures

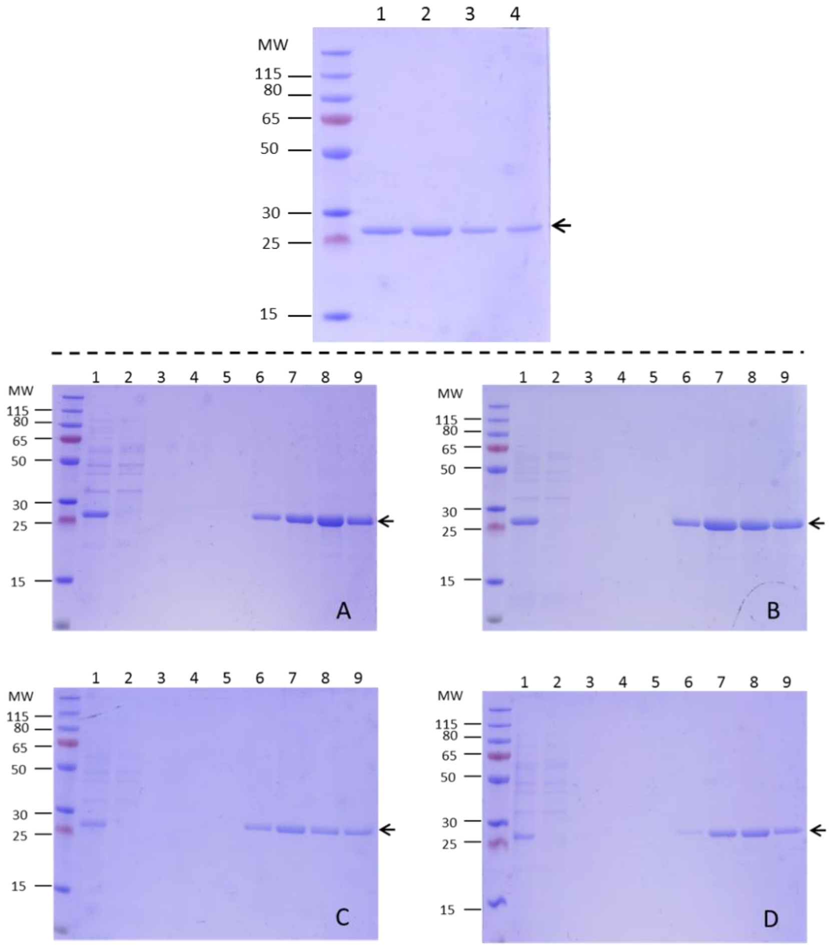

Supplementary Figure 1. FP engineering. Top: Overview of purified eGFP variants. Coomassie stained SDS-PAGE showing pooled eluate fractions (Ni-NTA affinity chromatography) after dialysis in PBS-buffer. Lane 1: eGFP-AA, lane 2: eGFP-FF, lane 3: eGFP-E222H, lane 4: eGFP wildtype. Samples were boiled in 4xLaemmli buffer prior to SDS-PAGE. MW stands for molecular weight in kDa. Proteins of interest are indicated by an arrow. Bottom: Purification of recombinant proteins *via* N-terminal 6xHis-Tag using Ni-NTA affinity chromatography under native conditions (non-denaturing). Coomassie stained SDS-PAGEs showing Ni-NTA purification procedure of (A) eGFP-AA, (B) eGFP-FF, (C) eGFP-E222H and (D) eGFP wildtype. Lane 1: supernatant after cell lysis and centrifugation step (soluble proteins), lane 2: column flow-through after incubation with Ni-NTA agarose (unbound proteins), lanes 3-5: washing steps, lanes 6-9: eluates (purified protein of interest). Samples were boiled in 4xLaemmli buffer prior to SDS-PAGE. MW stands for molecular weight in kDa. Proteins of interest are indicated by arrows.

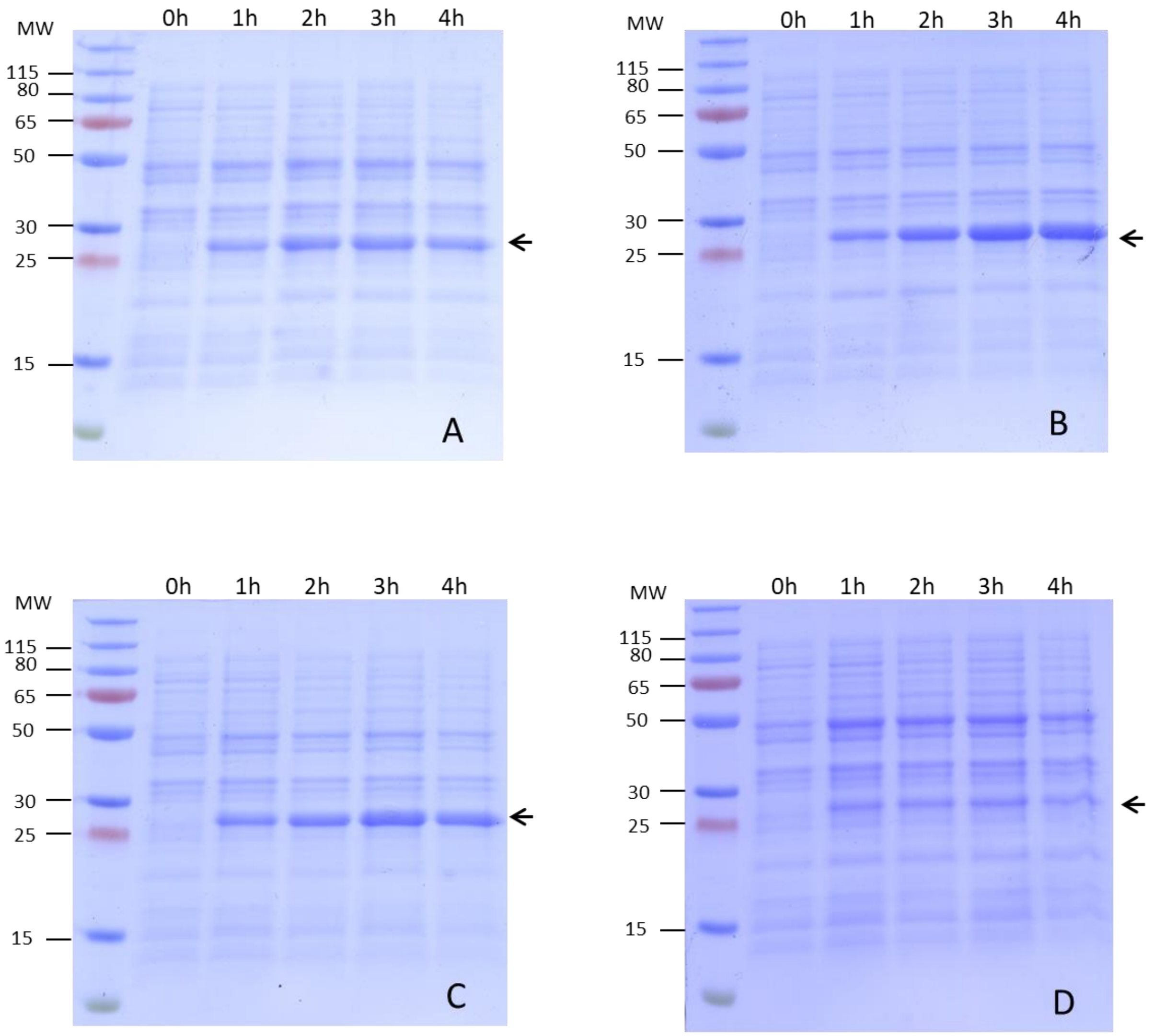

Supplementary Figure 2. FP engineering. Time course of heterologous expression of recombinant eGFP mutants and eGFP wildtype in *E.coli* M15 [pREP4] cells. Recombinant protein expression was monitored by SDS-PAGE and Coomassie Brilliant blue staining. Lane: 0h (before IPTG induction, no expression) , lane 2-5: 1h – 4h after IPTG induction (protein expression). Heterologous expression of (A) eGFP-AA, (B) eGFP-FF, (C) eGFP-E222H and (D) eGFP wildtype. Samples were adjusted to the same OD600. Samples were boiled in 4xLaemmli buffer prior to SDS-PAGE. MW stands for molecular weight in kDa. Proteins of interest are indicated by arrows.

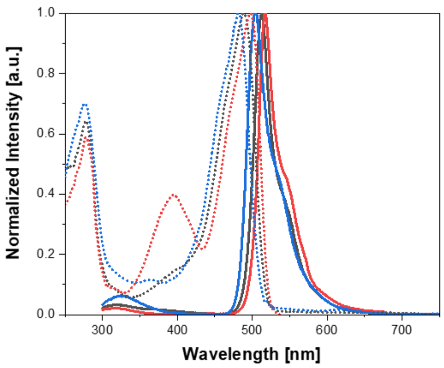

Supplementary Figure 3. FP spectroscopic characterization. Absorption (dashed line) and emission (solid line) spectra of eGFP (black), eGFP-FF (red), and eGFP-E222H (blue).

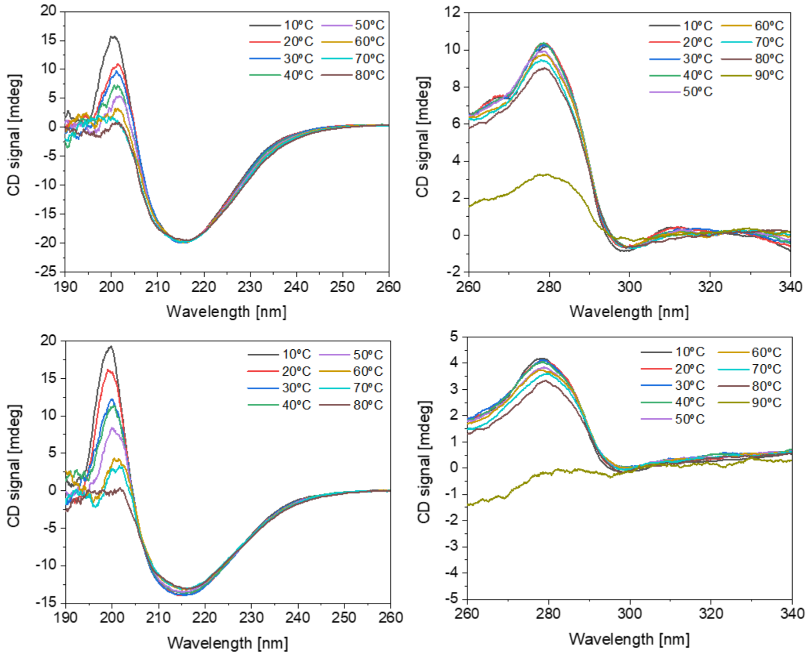

Supplementary Figure 4. FP spectroscopic characterization. Circular dichroism spectra characterization of secondary and tertiary structures of eGFP-FF (top) and eGFP-E222H (bottom).

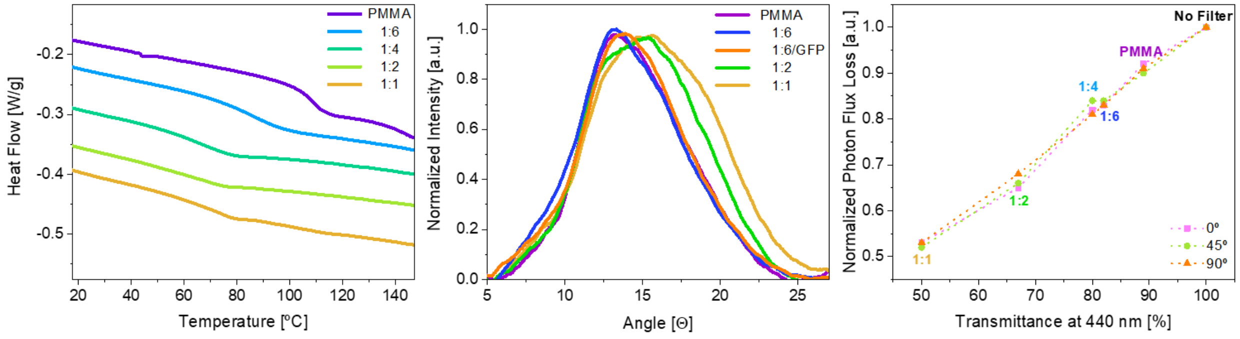

Supplementary Figure 5. Polymer composite characterization. MDSC (left), XRD (center), and photon flux loss of LED (440 nm) measured with respect to the vision angle (legend) and filter transmittance (right) of TMPE:PMMA films with differernt mass ratios.

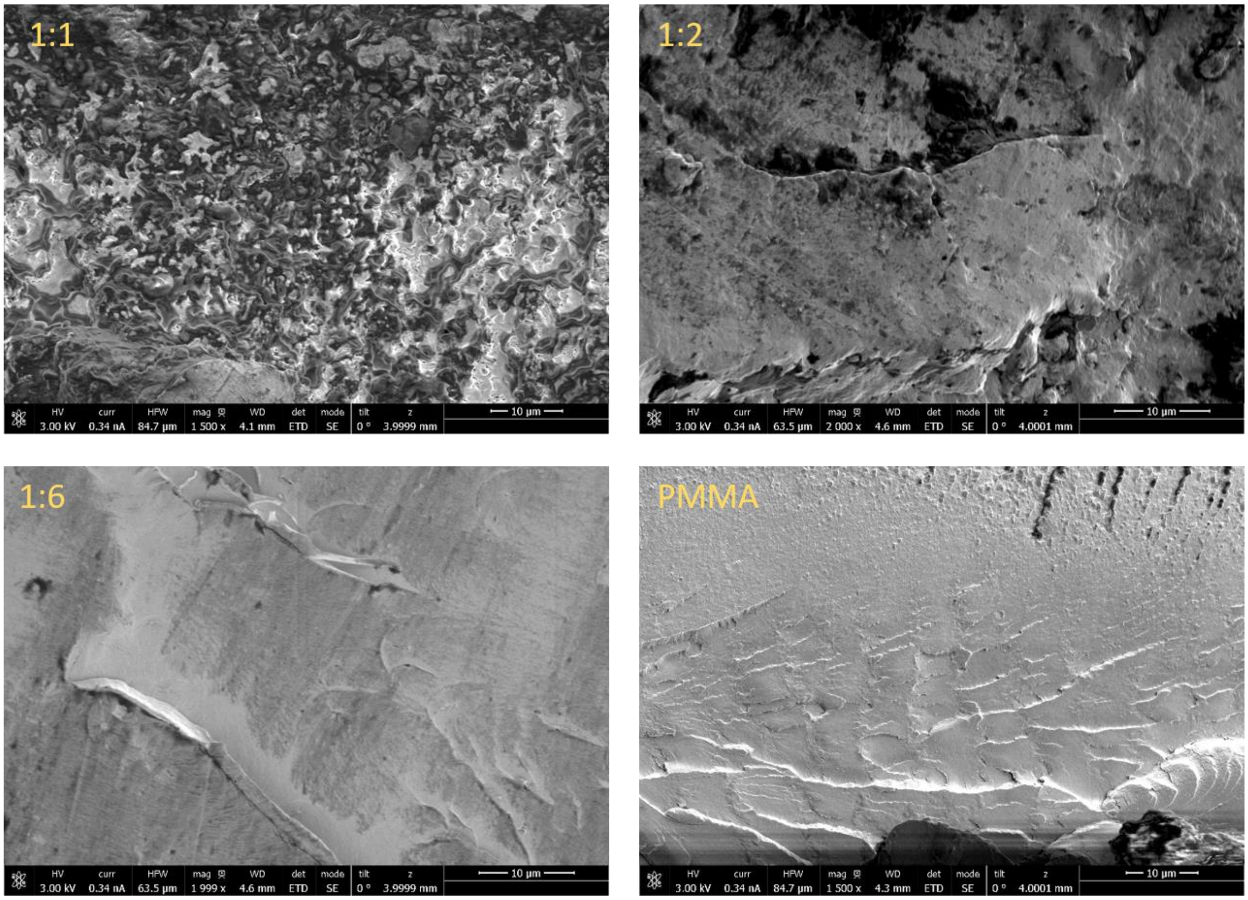

Supplementary Figure 6. Polymer composite characterization. Cross-sectional SEM images of 1:1, 1:2, 1:6, and pure PMMA films.

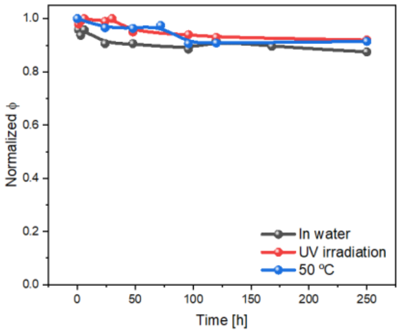

Supplementary Figure 7. Bio-phosphor characterization. Stability studies of the eGFP-AA 1:6 films at different stress scenarios – see legend.

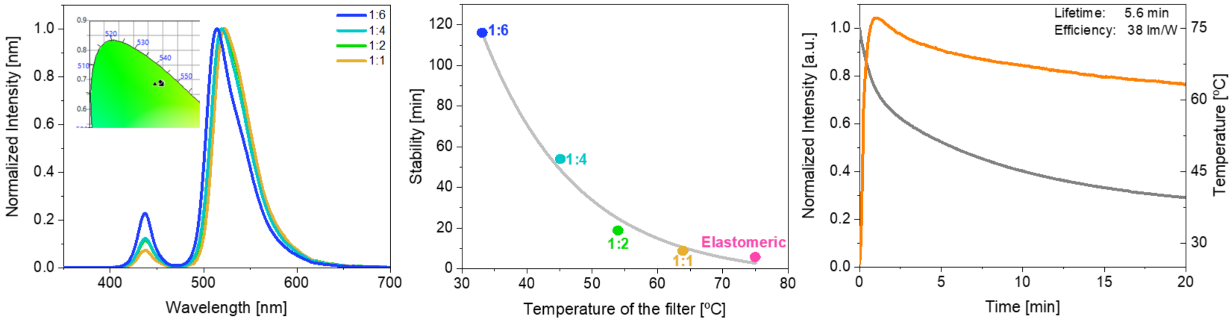

Supplementary Figure 8. Bio-HLED characterization. Left: Spectra of Bio-HLEDs with eGFP-AA doped TMPE:PMMA films with different mass ratios (see legend). Inset: x/y CIE color coordinates of the respective Bio-HLEDs. Center: Bio-HLED's stability *vs*. maximum temperature of the biophosphors. The fitting (grey line) highlights the exponential relation. Right: Intensity decay of the eGFP-AA emission (grey) and temperature rise of the filter (orange) of Bio-HLEDs with a reference elastomeric[1,2] filter containing the same amount of eGFP-AA.

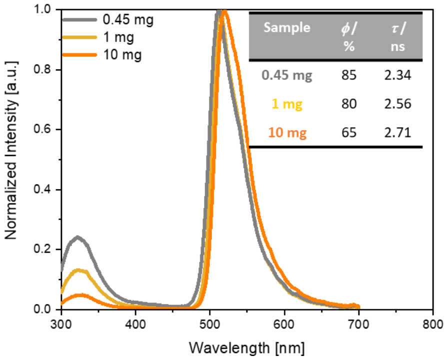

Supplementary Figure 9. Bio-HLED characterization. Emission spectra (λ_{exc} = 285 nm) of 1:6 films bearing different amounts of eGFP-AA (see legend). The inset table summarizes the ϕ and τ values for each film.

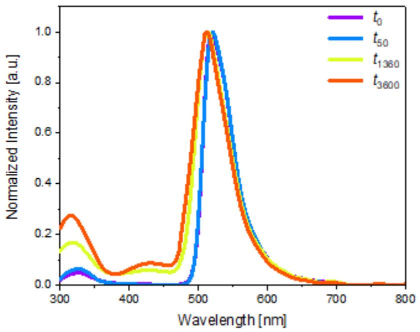

Supplementary Figure 10. Bio-HLED characterization. Emission spectra (λ_{exc}=285 nm) of the 1:6 color filters at selected times under device operating conditions.

2. References

1. Weber, M. D. *et al.* Bioinspired hybrid white light-emitting diodes. *Adv. Mater.* **27**, 5493–5498 (2015).

2. Niklaus, L. *et al.* Micropatterned down-converting coating for white bio-hybrid light-emitting diodes. *Adv. Funct. Mater.* **27**, 1–10 (2017).
